# Conventional and HD-tDCS May (or May Not) Modulate Overt Attentional Orienting: An Integrated Spatio-Temporal Approach and Methodological Reflections

**DOI:** 10.3390/brainsci12010071

**Published:** 2021-12-31

**Authors:** Lorenzo Diana, Giulia Scotti, Edoardo N. Aiello, Patrick Pilastro, Aleksandra K. Eberhard-Moscicka, René M. Müri, Nadia Bolognini

**Affiliations:** 1 Ph.D. Program in Neuroscience, School of Medicine and Surgery, University of Milano-Bicocca, 20900 Monza, Italy; e.aiello5@campus.unimib.it; 2 Department of Psychology, University of Milano-Bicocca, 20126 Milan, Italy; g.scotti13@campus.unimib.it (G.S.); patrick.pilastro@gmail.com (P.P.); 3 Perception and Eye Movement Laboratory, Departments of Neurology and BioMedical Research, Bern University Hospital Inselspital, University of Bern, 3010 Bern, Switzerland; aleksandra.eberhard@neuro.unibe.ch (A.K.E.-M.); rene.mueri@insel.ch (R.M.M.); 4 Department of Neurology, Bern University Hospital Inselspital, University of Bern, 3010 Bern, Switzerland; 5 Department of Psychology & Milan Center for Neuroscience (NeuroMI), University of Milano-Bicocca, 20126 Milan, Italy; nadia.bolognini@unimib.it; 6 Laboratory of Neuropsychology, Istituto Auxologico Italiano, IRCCS, 20122 Milan, Italy

**Keywords:** HD-tDCS, visuospatial attention, attentional orienting, eye tracking, free visual exploration

## Abstract

Transcranial Direct Current Stimulation (tDCS) has been employed to modulate visuo-spatial attentional asymmetries, however, further investigation is needed to characterize tDCS-associated variability in more ecological settings. In the present research, we tested the effects of offline, anodal conventional tDCS (Experiment 1) and HD-tDCS (Experiment 2) delivered over the posterior parietal cortex (PPC) and Frontal Eye Field (FEF) of the right hemisphere in healthy participants. Attentional asymmetries were measured by means of an eye tracking-based, ecological paradigm, that is, a Free Visual Exploration task of naturalistic pictures. Data were analyzed from a spatiotemporal perspective. In Experiment 1, a pre-post linear mixed model (LMM) indicated a leftward attentional shift after PPC tDCS; this effect was not confirmed when the individual baseline performance was considered. In Experiment 2, FEF HD-tDCS was shown to induce a significant leftward shift of gaze position, which emerged after 6 s of picture exploration and lasted for 200 ms. The present results do not allow us to conclude on a clear efficacy of offline conventional tDCS and HD-tDCS in modulating overt visuospatial attention in an ecological setting. Nonetheless, our findings highlight a complex relationship among stimulated area, focality of stimulation, spatiotemporal aspects of deployment of attention, and the role of individual baseline performance in shaping the effects of tDCS.

## 1. Introduction

In the last decades, we have witnessed a rise in the application of non-invasive brain stimulation (NIBS) techniques to modulate cognitive functions both in healthy individuals and clinical populations [1]. Among these techniques, transcranial direct current stimulation (tDCS) has received great attention thanks to its ease of use, low costs, and tolerability. tDCS is based on the application of a weak electrical current on the participant’s scalp, delivered by means of two electrodes, that is, the anode and the cathode. Several neurophysiological studies showed that tDCS can exert polarity-dependent, long-lasting changes of membrane resting excitability [2,3] by modulating neural plasticity mechanisms [4] and altering long-range connectivity of functionally connected areas [5].

Despite its acknowledged potential, tDCS is often associated with large inter-individual variability of responses and scarce reproducibility of results [6]. These findings are likely due to multiple interacting factors. For example, tDCS experiments are often statistically low-powered with small sample sizes. Efforts should be made to estimate adequate sample sizes in light of the medium-to-small effect sizes associated with this technique [7]. Moreover, behavioral and neurophysiological tDCS effects have been shown to depend on the state of brain activation during the stimulation (see [8] for a theoretical perspective) and, accordingly, on individual neurophysiological and behavioral baseline levels. For instance, participants showing higher performance at a given cognitive task may not benefit from tDCS as much as “lower” performers (e.g., [9]). Lastly, tDCS has been conventionally delivered by means of large rubber electrodes, normally sized 25–35 cm^2^, with an electrode placed over the target region and a return electrode placed either on a cephalic or extracephalic area. Current-modeling studies have highlighted the low spatial specificity of this approach and indicated that the electric field may widely spread and peak over unintended areas [10]. In an attempt to reduce current spread and control for the large variability reported in tDCS experiments, more focal electrodes and montages were introduced, the so-called High Definition tDCS (HD-tDCS). HD-tDCS exploits a smaller target electrode usually surrounded by four return electrodes (i.e., 4 × 1 montage) [10] or a ring-shaped return electrode [11]. Compared to conventional tDCS, HD-tDCS applied to the motor cortex was shown to induce longer-lasting after-effects, peaking 30 min after the stimulation [12]. However, the efficacy and the timing of HD-tDCS delivered over associative cortices, in the context of cognitive tasks, require further investigation.

In the present study, we investigated the behavioral outcomes of tDCS and its variability in the context of visuospatial attention in young healthy participants; more specifically, we aimed to test the effects of conventional tDCS and HD-tDCS on spatial attentional asymmetries.

Several studies applied tDCS to modulate visuospatial orienting and lateralized biases, both in healthy volunteers and patients with acquired visuospatial disorders [13,14,15]. In general, the theoretical background of this approach is based on the concept of interhemispheric competition between two attentional vectors, each directed to the contralateral hemispace [16]. Accordingly, an increase (or decrease) of excitability of one hemisphere should alter this balance, thus biasing attention towards the contralateral (or ipsilateral) hemisphere. The balance of attentional weights across the hemispheres relies on the activity of fronto-parietal attention networks [17], whose dorsal component, including the Frontal Eye Field (FEF) and different regions of the posterior parietal cortex (PPC), are bilaterally represented [18].

So far, most of the research conducted on healthy participants focused on the effects of parietal stimulations in a wide range of computerized tasks. For example, Sparing et al. [13] found that 10 min of 1 mA anodal or cathodal offline stimulation of the right PPC enhances (anodal) or decreases (cathodal) the detection of contralateral visual targets. Giglia and co-workers [19] applied 15 min of 1 mA online cathodal tDCS over right PPC or bihemispheric parietal tDCS (cathode right PPC–anode left PPC) and reported a “neglect-like” rightward bias in a landmark task. Moreover, in a multisensory detection task, Bolognini and colleagues [14] found that 15 min of 2 mA anodal stimulation speeded up the detection of contralateral visual targets. Notably, some authors also applied tDCS to frontal areas [20,21]. Roy and colleagues [21], in a modified Attention Network Task, reported that anodal tDCS over the right PPC improved the processing of contralateral targets, especially when attention had to be re-oriented rightwards. No effects of left anodal tDCS over the dorsolateral prefrontal cortex (DLPFC) were found. In a conjunction search task, Ball et al. [20] found no effects of anodal tDCS delivered over the right PPC or the right FEF.

Besides classical computerized tasks, visuospatial attention can be investigated by means of eye movement recording. In everyday life, attention is directed “overtly” most of the time. Neural circuits of spatial attention and eye movements share common neural underpinnings [22,23,24], although dissociable under some circumstances [25,26]. The results of the few tDCS studies on eye movements, which applied tDCS over the FEF to modulate saccadic latency towards lateralized targets, reported contradictory findings [27,28]. More recently, Diana and colleagues [29] investigated the effects of anodal tDCS over the right PPC and FEF in a saccadic paradigm (i.e., the gap-overlap paradigm) to study orienting and disengagement of visuospatial attention. Besides a small, bilateral improvement of saccadic latency following stimulations of both target regions, the authors reported significant effects of the baseline performance on tDCS-induced effects.

An interesting paradigm to study the deployment of visuospatial attention is the free visual exploration task (FVE) [30,31]. The FVE task is a simple, ecological, eye-tracking-based paradigm in which participants are asked to freely explore images of everyday scenes. In general, the analysis of the distribution of fixations (e.g., the average position of fixations on the left-right axis or the amount of time spent on the left or the right side of the images) provides informative indexes of spatial exploration and asymmetries. Indeed, the FVE task has been employed to characterize exploration patterns of brain-damaged patients with hemispatial neglect [32], showing to be a sensitive measure for assessing the typical attentional asymmetries [33,34]. Moreover, the FVE task has been also administered to healthy volunteers to study the phenomenon of pseudoneglect [30,35,36]. Within the field of NIBS, FVE was used with transcranial magnetic stimulation (TMS) to assess the role of FEF in overt visual exploration, demonstrating a bilateral involvement of the right FEF in this process [37]. Overall, the FVE task is a robust paradigm to investigate the overt allocation of visuospatial attention ecologically. Additionally, FVE offers the opportunity to characterize visuospatial exploration from a temporal perspective, that is to investigate how spatial asymmetries change over the exploration time. For instance, Chiffi and colleagues [31], by adopting this spatiotemporal approach, found a time-sensitive, age-dependent, modulation of pseudoneglect in the FVE task which correlated with the individual performance at a line bisection task.

In the present study, we took advantage of the FVE task, adopting the spatiotemporal approach, to investigate the behavioral effects of anodal conventional tDCS (Experiment 1) and HD-tDCS (Experiment 2) on visuospatial exploration. Specifically, we addressed the following questions:

(1) Does the modulation of right frontal (FEF) and parietal (PPC) areas induce contralateral, leftward attentional shifts et in a more ecological task? Are these effects different depending on the stimulated area?
(2) Considering the reduced current spread, does HD-tDCS induce more consistent effects than conventional tDCS? Do its aftereffects emerge at different times post-stimulation (e.g., [12])?

## 2. Experiment 1—Conventional tDCS

### 2.1. Materials and Methods

#### 2.1.1. Participants and Sample Size Estimation

As we expected a significant level of interindividual variability in the response to tDCS, we planned to analyze tDCS effects by means of mixed models. Although different methods to estimate sample size for mixed models exist [38], it is challenging to retrieve all needed parameters for a proper a priori analysis because previous works on tDCS and visuospatial attention did not employ this statistical approach. Therefore, the sample size was estimated for both Experiment 1 and Experiment 2 (see below) with an a priori power analysis [G*Power 3.1.9.6; Heinrich-Heine-Universität Düsseldorf, Düsseldorf, Germany] for a repeated-measures analysis of variance (ANOVA), assuming it may yield a sufficient approximation of the needed sample size. As suggested by Minarik et al. [7], we specified a medium-small effect size, that is, *f* = 0.2 (*d* = 0.4), α = 0.05, 1−β = 0.08, correlation among measures = 0.05, sphericity correction = 1, and number of measurements = 6, as each participant carried out the experimental task six times (i.e., before and after tDCS in three different sessions). Dropout risk was not considered. The analyses indicated 28 participants. Three recruited participants were excluded because of eye-tracking technical problems but were replaced to meet the required sample size. The final sample included 18 females and 12 males (mean age = 25 ± 3 years), all recruited at the University of Milano-Bicocca. Inclusion criteria for the study were: right-handedness according to the Edinburgh Handedness Inventory [39], normal or corrected-to-normal visual acuity, and absence of contraindications to tDCS [40,41]. The study was approved by the Ethics Committee of the University of Milano-Bicocca (Protocol 457–27 November 2019) and was conducted in accordance with the ethical standards of the Declaration of Helsinki. All participants provided their written informed consent.

#### 2.1.2. Free Visual Exploration (FVE) Task

The task consisted of the free exploration of sets of 12 naturalistic pictures (1680 × 1050 pixels, 35° × 22° of visual angle, presentation time = 7 s); each picture was preceded by a black screen with a fixation cross lasting for 1.5 s. As the participants performed the task six times (i.e., before and after tDCS in three different sessions; see below), different pictures, but comparable in terms of left and right saliency, were presented in each experimental session. Specifically, a total of 108 pictures were selected from a previously used database [31,36]. A saliency matrix based on several features such as orientation, color, and intensity was obtained for each picture with a MATLAB (R2019b) algorithm [42]. Subsequently, the ratio between mean left-right saliency was calculated and pictures were randomly assigned to nine different blocks of pictures. A one-way ANOVA confirmed no significant difference between blocks in terms of mean left-right saliency ratio (F_899_ = 0.78; *p* = 0.618). Therefore, each participant was randomly assigned six out of nine possible blocks. Moreover, to further reduce the impact of pictures content on visuospatial asymmetries, the same blocks were mirrored along the vertical midline for half of the participants. Pictures and their respective saliency values are available on Open Science Framework (OSF) at: https://osf.io/4sdwr/ (accessed on 7 November 2021).

The FVE task was performed in a dark room. Participants were seated in front of a monitor (Acer HN274H 27″) aligned with their mid-sagittal plane at a viewing distance of 83 cm; the alignment was kept constant employing a chin-and-head rest. Eye movements were recorded by means of an EyeLink 1000 (SR Research Ltd., Kanata, ON, Canada). At the beginning of the task, the eye tracker was calibrated using a nine-point grid and the mean gaze accuracy was kept, on average, around 0.5° of visual angle. The experiment was programmed with SR Research Experiment Builder 2.3.1 (SR Research Ltd., Ottawa, ON, Canada).

#### 2.1.3. tDCS Protocol and Experimental Procedure

**Figure 1:**
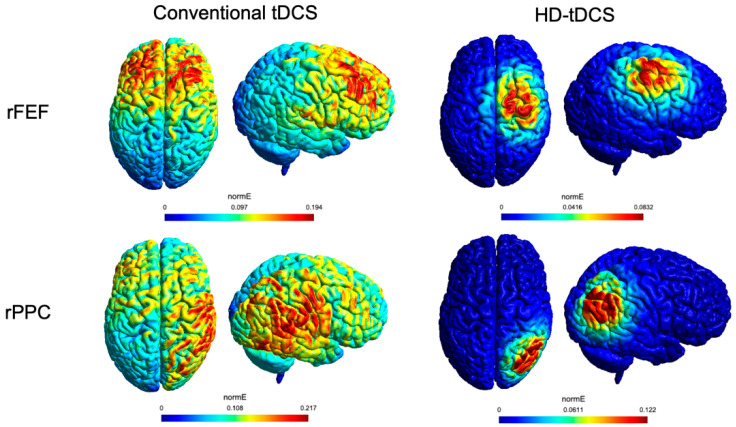
Simulation of the electric field (normE-V/m) performed with SimNIBS. Side and top views of the right posterior parietal cortex (rPPC) and the right frontal eye field (rFEF) are provided for conventional tDCS (left panel) and HD-tDCS (right panel).

tDCS was delivered by a battery-driven current stimulator (BrainSTIM device, E.M.S., Bologna, Italy) through two electrodes inserted into saline-soaked sponges (target electrode: 5 × 5 cm^2^ and reference electrode: 7 × 5 cm^2^). Anodal tDCS was applied to the target areas (i.e., right FEF and right PPC) for 10 min at 1 mA intensity (^13^), with 10 s fade-in and fade-out. In the case of sham tDCS, the stimulator was turned off after 30 s (i.e., 10 s fade-in, 10 s of stimulation, 10 s fade-out). The protocol was single-blind: whereas participants were kept blind to the tDCS condition (i.e., real or sham), the experimenters were not. Each participant underwent three experimental tDCS sessions over three different days. In each session, tDCS was applied over the right FEF, over the right PPC, or it could be delivered as sham stimulation (the sham tDCS was applied over the FEF for half of the participants, and for the other half over PPC). The order of the three experimental tDCS sessions was counterbalanced across participants. Target areas were marked on an elastic cap that was centered on participants’ heads. FEF and PPC had been previously identified by means of a neuronavigation procedure (Softaxic 2.0, E.M.S., Bologna, Italy) on 10 healthy volunteers. The stereotaxic MNI coordinates were: 44, −66, 43 for right PPC (corresponding to P4 of the 10–20 system) [43] and 23, −13, 59 for right FEF [44]. The anode was placed over the right FEF or PPC (at the center of the 10 marks identified through the neuronavigation system) depending on the condition, whereas the reference electrode (i.e., cathode) was located over the left forehead, in a supraorbital position. The electrodes were fixed on the scalp with two elastic bands. Figure 1 depicts a simulation of the induced electrical field, calculated with SimNIBS 3.2 [45]. More details about the procedure are reported in Appendix A.

During each experimental session, participants performed the FVE task before the beginning and after the end of tDCS. During the stimulation, they were asked to relax and look at a blank screen. The sessions took place at the same time of the day, separated by at least 24 h to avoid any possible carry-over effects. At the end of each session, a questionnaire was administered to collect sensations experienced during tDCS [46]. At the end of the last session, participants were also asked to report whether they received real or placebo stimulations and when. Overall, tDCS was well tolerated and no serious adverse effects occurred. The most-reported sensation was head itching of mild intensity, which began at stimulation onset and quickly stopped. In general, the total score at the questionnaire was higher for both PPC tDCS (*p* = 0.013) and FEF tDCS (*p* = 0.032), as compared to sham tDCS. With respect to the blinding to stimulation, only 7 out of 28 participants correctly identified all three stimulations (see Appendix B for more details).

### 2.2. Data Analyses

Eye movements were automatically parsed into fixations and saccades according to the eye-tracking manufacturer’s standard thresholds for velocity and acceleration, that is, 30°/s and 8000°/s^2^. This setup is reported to be the best for cognitive research, as it reduces the number of microsaccades and the number of short fixations (<100 ms). Fixation parameters were computed and exported with the SR Research Data Viewer software (SR Research Ltd., Ottawa, ON, Canada). After the exclusion of fixations falling outside of the picture area (0.35%), the final dataset included 54,163 fixations, with a mean number of fixations per image of 22.2 (SD = 3.9; range = 12–28).

Data processing, analysis, and visualization were performed by using R 3.6.2 [47] and specific packages [48,49,50,51,52,53] in R-Studio 1.2.5033 [54]. Datasets are available at https://osf.io/4sdwr/ (accessed on 7 November 2021).

To analyze the effects of tDCS on the FVE pattern, we adopted an integrated spatiotemporal approach. Previous research [31,35,36] showed a characteristic time-course of visual asymmetries during free visual exploration characterized by an initial exploration of the left side of the pictures—interpreted as pseudoneglect—followed by the exploration of the right side with a final return towards the center of the picture. Here, we aimed to investigate whether and when could tDCS modulate such a typical free visual exploration pattern.

To this aim, firstly we tested the effects of tDCS on the average horizontal fixation position, irrespective of the viewing time; then, we employed a non-parametric random permutation procedure to get insights into the effect of time [36,55].

#### 2.2.1. Can tDCS Induce a Contralateral Attentional Shift?

In order to investigate left-right asymmetries, we calculated the mean fixation position on the horizontal axis in pixels—henceforth, the average gaze position–for each participant, considering as main factors: Stimulation (FEF, PPC, and sham) and Timepoint (baseline—T0 and post-tDCS—T1). Moreover, as some participants explored the pictures more actively than others (i.e., more saccades/shorter fixations vs. fewer saccades/longer fixations), the mean number of fixations for each Stimulation and Timepoint were also analyzed.

As a first step, we followed a classic “pre-post” approach, that is the comparison between the baseline (T0) and the post-tDCS (T1) performances for each Stimulation. A Linear Mixed Model (LMM) was used, with the average gaze position as a dependent variable; fixed effects were tested for Stimulation (FEF, PPC, and sham), Timepoint (T0 and T1), their interaction, and the mean number of fixations. Random intercepts were allowed for Stimulation and Timepoint. The significance of the fixed effects was evaluated by means of F-tests with Satterthwaite’s method.

Afterward, the effects of tDCS were investigated by means of a baseline-dependent analysis. Indeed, it has been reported that pre-post approaches, as well as correlational ones between the baseline and change of performance, may overlook some potential statistical issues such as the regression to the mean [56,57]. Therefore, we ran an LMM with the average gaze position after tDCS (at T1) as the dependent variable; fixed effects were tested for Stimulation (FEF, PPC, and sham), with the baseline performance as covariate (i.e., the average gaze position at T0), and the interaction between Stimulation and the baseline. The mean number of fixations was not included in this analysis because there was no effect in the previous model (See Results). Random intercepts were allowed for participants only because the addition of “Stimulation” yielded a singular fit.

In every analysis, the significance of the fixed effects was assessed by means of F-tests with Satterthwaite’s method and significant interactions were explored by means of post-hoc comparisons with Bonferroni correction.

#### 2.2.2. The Effects of tDCS on Visual-Exploration Patterns from a Temporal Perspective

In order to investigate when (i.e., at which time of the 7 s time window of exploration) tDCS has an effect, we adopted a similar approach as in Chiffi et al., 2021 [31]: each fixation’s starting time from picture presentation was converted into time-bins of 100 ms (i.e., 70 possible time-bins for 7 s of picture duration). Then, the average gaze position (i.e., the mean fixation position on the horizontal axis) was calculated for each participant, stimulation session (FEF, PPC, and sham), timepoint (T0 and T1), and time-bins. For each time-bin, we tested whether the interaction between Stimulation and Timepoint was a significant predictor of the gaze position. To account for the problem of multiple comparisons, we implemented the non-parametric random permutation procedure by Maris and Oostenveld [58]. Accordingly, adjacent 100 ms bins found significant for a predictor (*p* < 0.05) formed a cluster; Fisher’s F values of all bins within a cluster were summed, resulting in “cluster mass values”. These values were then compared to a “random distribution” of mass values obtained from randomly permutated bins 5000 times. Stimulation and Timepoint were included as random terms. Permutations, as well as the corresponding *p* values, were obtained using the R-package permuco (53). Finally, in the case of a significant time cluster for the Stimulation by Timepoint interaction, the horizontal gaze position was averaged for the significant time clusters and an LMM was run with Stimulation × Timepoint as a factor, and random intercepts for Stimulation and Timepoint. The significance of the fixed effects was assessed by means of F-tests with Satterthwaite’s method and significant interactions were explored by means of post-hoc comparisons with Bonferroni correction.

### 2.3. Results

**Figure 2:**
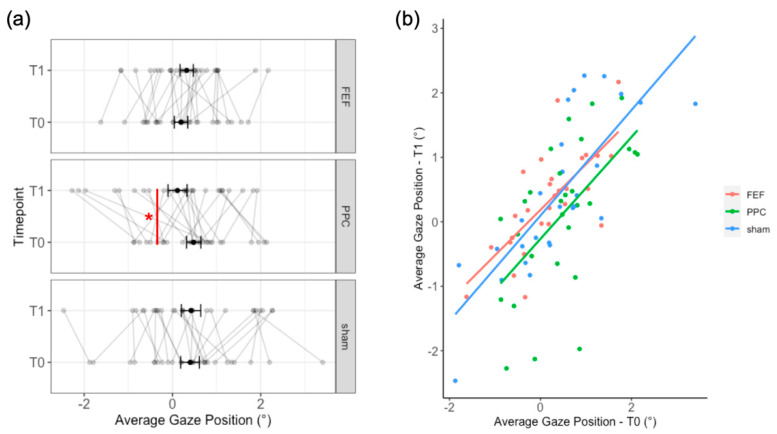
(**a**) Average gaze position relative to the center of the picture for each stimulation session and timepoint. Bold black lines represent the mean values with the standard errors, while individual performances are depicted with grey lines. The red line represents the significant post-hoc comparisons (pre-post LMM). After PPC tDCS (T1), as compared to its baseline (T0), participants’ gaze position shifted slightly to the left. (**b**) Regression lines for each stimulation on the baseline performance, as from the “baseline” LMM; * = *p =* 0.024.

The “pre-post” LMM yielded a significant Stimulation by Timepoint interaction (*F*_258.8_ = 3.91; *p* = 0.026). Post-hoc comparisons showed a small leftward deviation (17.7 pixels, 0.37° of visual angle, *t* = 2.55, *p* = 0.025) after PPC stimulation as compared to its baseline. No effect of the mean number of fixations was found (*F*_1118.4_ = 1.9; *p* = 0.17). Full results are reported in Appendix B and illustrated in Figure 2a.

The “baseline” LMM showed no effect of Stimulation on the average gaze position after tDCS (*F*_260.34_ = 0.19; *p* = 0.825). We found a significant effect of the baseline (*F*_166.04_ = 52.98; *b* = 0.073; *p* < 0.001), indicating a positive association with the post-tDCS gaze position. No interaction between the baseline and Stimulation was observed (*F*_260.38_ = 0.2; *p* = 0.82; see Figure 2b for a graphical representation of the baseline effect).

The non-parametric random permutation revealed no significant clusters of time-bins for Stimulation, Timepoint, or their interaction. Therefore, we did not conduct any further analysis. More details are reported in Table A3 of Appendix B.

### 2.4. Discussion

In this first experiment, we tested the effects of conventional tDCS over the right PPC or the right FEF on overt orienting of visuospatial attention. We investigated whether anodal tDCS, which is thought to increase cortical excitability, could induce a contralateral shift of the attentional focus, measured with the FVE task. We adopted a spatio-temporal perspective to unravel not only whether but also when (i.e., at which moment of the visual exploration) tDCS modulated visuospatial orienting.

Interestingly, the first LMM, named the “pre-post” model, indicated a very small (around 17 pixels, i.e., 0.37° of visual angle) leftward shift of the average gaze position only after PPC stimulation, but not after FEF or sham tDCS. However, the non-parametric random permutation approach did not reveal a particular time window where this effect occurred, suggesting it may be different across participants. Overall, these results complement previous evidence [13,14,21] showing a benefit for attentional orienting towards contralateral stimuli brought about the anodal stimulation of the right PPC. These findings are in line with the model of interhemispheric rivalry by Kinsbourne [16], according to which an increased activity of the right-hemisphere vector (achievable by means of anodal tDCS) would increase its inhibitory effect over the left hemisphere, in turn biasing attention towards the left hemispace.

However, a different conclusion may be drawn by analyzing the same data with a different statistical approach. Indeed, when the baseline performance was considered to predict tDCS effects, no modulation of gaze position was found after tDCS. Several studies have previously shown the impact of the level of baseline performance and of the brain activation state on tDCS efficacy [9,57,59]. Here, we found a positive association between the baseline attentional bias (leftward or rightward) and post-tDCS performances, but regardless of the type of stimulation (real or sham) and of the target area (FEF, PPC), indicating overall stability of gaze position across stimulation sessions, as well as before and after tDCS. However, by using the pre-post gaze position change (T1 *minus* T0) in a second model (see Appendix A), the relationship becomes negative, suggesting a reversal of the attentional bias post-tDCS: those participants with rightward bias at baseline were more likely to show a leftward shift of gaze after tDCS, and vice versa. It is worth mentioning that using the baseline performance to predict a score change often leads to negative associations [56] and even distorted results because of statistical phenomena, such as the regression to the mean. Indeed, some authors [57] suggest that the best approach should be the one we followed in the “baseline” model.

Moreover, as observed by inspecting individual data (see Figure 2a), performance at the FVE task appears quite variable both before and after tDCS. This task has an intrinsic variable nature since the participant is free to explore the picture by following their own personal strategy or preference. One could argue that any observable change could be merely due to such variability. Nonetheless, we controlled for intrinsic picture variability by creating blocks of picture balanced for left-right saliency and by mirroring these blocks in half of the participants. Furthermore, since some participants may employ more active exploration strategies, rather than spending more time on single elements of the picture, we included the mean number of fixations in the first LMM, but no effect was found.

A critical factor potentially explaining the present findings is the low spatial resolution of the conventional tDCS (see Figure 1). Therefore, in Experiment 2, we employed HD-tDCS with concentric electrodes [11,60] to explore whether: (1) a more focal stimulation could induce more specific and reliable effects on visuospatial asymmetries; (2) the HD-tDCS effects could emerge later, by adding a third timepoint (i.e., T2) 30 min after the end of the stimulation [12]; (3) the HD-tDCS effects could depend on the viewing time (i.e., random permutation analyses on the time bins).

## 3. Experiment 2—HD-tDCS

### 3.1. Materials and Methods

#### 3.1.1. Participants

For sample size estimation, we used the same procedure as in Experiment 1 (see Section 2.1.1), but the number of measurements was set to nine, as participants carried out the experimental task nine times (i.e., three timepoints in three stimulation sessions, see Section 3.1.3 for more details). According to the results, we recruited 22 right-handed participants (14 females, mean age = 23 ± 3 years); none of them dropped out. They all complied with inclusion criteria for NIBS and provided their written informed consent.

#### 3.1.2. FVE Task

Task parameters and experimental setting were the same as for Experiment 1, except that we created nine new blocks of 25 pictures, for a total of 225 pictures: 120 pictures were taken from the database already used in Experiment 1 and 115 new pictures of naturalistic and urban landscapes were downloaded from Pixabay.com (accessed on 7 November 2021). The choice of increasing the number of pictures was motivated by the high variability of subjects’ performance observed in Experiment 1. In the attempt to reduce such variability, in Experiment 2 we balanced blocks presentation across experimental sessions and timepoints and doubled the number of pictures in each block (i.e., 25 pictures). A one-way ANOVA confirmed the absence of between-block difference in the ratio between left and right saliency (*F*_8216_ = 0.87; *p* = 0.547). Block order was counterbalanced across participants and every block was presented at least once for each combination of Stimulation and Timepoint. Moreover, half of the participants were administered the same combination of blocks in the same order, but pictures were mirrored along the vertical midline.

#### 3.1.3. HD-tDCS Protocol and Experimental Procedure

The tDCS protocol was the same as for Experiment 1 in terms of intensity, duration, and localization of target areas. Now, electric current was delivered through two concentric rubber electrodes (neuroCare Group AG, Munich, Germany), a round target electrode (Ø = 25 mm), and a reference ring electrode (outer Ø = 100 mm, inner Ø = 75 mm) positioned around the target one. This kind of configuration was first used by Bortoletto et al. [11] for motor cortex stimulation, showing a high focality. Compared to 4 × 1 HD-tDCS setups, a setup with concentric electrodes is more affordable and compatible with classical stimulators. To reduce impendence, ten20 paste (Weaver and Co., Aurora, CO, USA) was applied to both electrodes. Moreover, an elastic tubular net kept the electrodes in place. Overall, the most reported sensation was head itching of mild intensity, which began at stimulation onset and quickly stopped. Moreover, more frequent and intense sensations were associated with PPC HD-tDCS, as compared to sham stimulation (*p* = 0.048). Full results from the questionnaires assessing the sensations experienced during the stimulation and the sham blinding are reported in Appendix C.

The experimental procedure was the same as that of Experiment 1, but we included a third assessment (i.e., T2) given the FVE task 30 min after the end of the stimulation.

#### 3.1.4. Data Analyses

Analyses were conducted on 109,267 fixations, excluding those outside of the picture perimeter (i.e., 0.26%). The mean number of fixations per image was 22.1 (SD = 4.5; range = 12–31). Datasets are available at https://osf.io/4sdwr/ (accessed on 7 November 2021).

The same statistical approach of Experiment 1 was adopted. Firstly, we performed a “pre-post” LMM with the average gaze position as the dependent variable, predicted by the Stimulation (FEF, PPC, and sham) by Timepoint (T0, T1, T2) interaction, and by the mean number of fixations. Random intercepts were calculated for participants only.

Subsequently, we ran a “baseline” LMM on the average gaze position after tDCS. Fixed effects were tested for the interaction between Stimulation (FEF, PPC, and sham) and Timepoint (T1 and T2) and for the baseline performance. Random intercepts were allowed for participants. The interactions with the baseline performance and Stimulation were decomposed by analyzing the simple effects.

By using a non-parametric random permutation procedure, we tested for each time-bin of 100 ms the Stimulation (FEF, PPC, and sham) × Timepoint (T0, T1, T2) interaction. In the case of significant time clusters for such interaction, the horizontal gaze position was averaged for the time period of the significant clusters. An LMM was used with Stimulation by Timepoint as factor and random intercepts for participants.

In every analysis, the significance of the fixed effects was evaluated by means of F-tests with the Satterthwaite method, whereas significant main effects or interactions were explored with Bonferroni-corrected post-hoc comparisons.

### 3.2. Results

**Figure 3:**
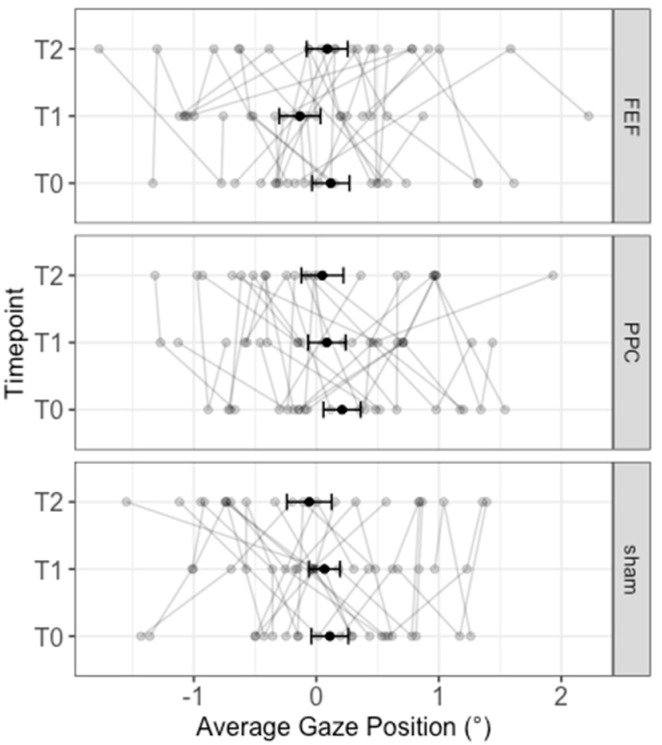
Average gaze position relative to the center of the picture for each stimulation and timepoint. Bold black lines represent the mean values and the standard errors. Individual performances are depicted with grey lines.

Overall, the “pre-post” LMM showed no significant effect: Stimulation (*F*_2167.66_ = 0.59; *p* = 0.558), Timepoint (*F*_2167.78_ = 1.29; *p* = 0.28), Stimulation × Timepoint (*F*_4167.78_ = 0.61; *p* = 0.659), average number of fixations (*F*_1116.72_ = 0.81; *p* = 0.37). Results are reported in Figure 3.

Likewise, the “baseline” LMM yielded no significant effects of Stimulation, Timepoints, the baseline, or their interactions (all *ps* > 0.101; see also: Appendix C, Table A6).

**Figure 4:**
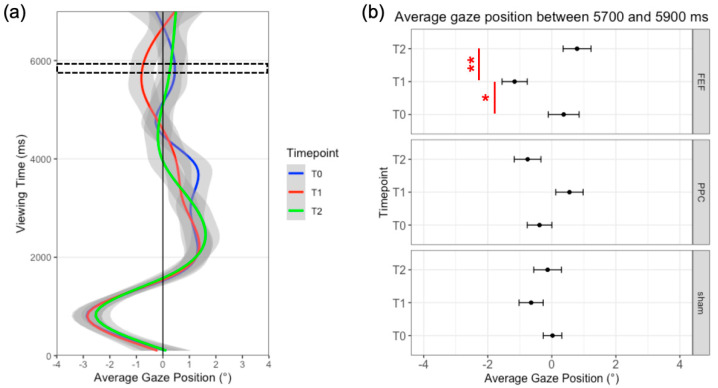
(**a**) Time-course of visual exploration at different timepoints for FEF HD-tDCS. The black dotted box represents the time window of interest between 5700 and 5900 ms. (**b**) Average gaze position relative to the center of the picture, between 5700 and 5900 ms, for each stimulation and timepoint. Red lines represent significant differences. Error bars= standard error. * = *p* < 0.05; ** = *p* < 0.001.

Interestingly, the temporal analysis on time-bins (i.e., non-parametric permutations) revealed a small significant cluster of 200 ms (i.e., between 5700 ms and 5900 ms, cluster mass = 9.46; *p* = 0.005) during which the interaction between Stimulation and Time reached the significance. The LMM calculated on the mean fixation position within this time-frame, indeed, showed a significant Stimulation x Timepoint interaction (*F*_4168_ = 5.07; *p* < 0.001): a larger leftward bias was found after FEF stimulation (i.e., T1) as compared to the baseline (T0 = −37.58 pixels; −0.78° of visual angle; *t* = −2.54; *p* = 0.036) and the 30-min assessment (T2 = −55.26 pixels; 1.15° of visual angle; *t* = −3.74; *p* < 0.001). No significant differences emerged for sham and PPC tDCS (all *ps* > 0.05). Results are shown in Figure 4.

### 3.3. Discussion

The results of Experiment 2 showed that HD-tDCS did not modulate the gaze position (without the viewing time), not confirming the trend observed in Experiment 1 with conventional tDCS, that is, induction of a leftward shift after PPC stimulation. Nevertheless, a more fine-grained analysis of the time-bins revealed that participants showed a leftward shift of attention around 5800 ms after FEF stimulation (T1), as compared to the baseline performance (T0) and the performance after 30 min from the end of the same stimulation (T2).

These findings could be driven by a tDCS-induced up-regulation of the dorsal frontoparietal network, which includes the FEF area, mediating top-down attentional orienting [17]. Indeed, this effect occurs at a later stage of the exploration behavior, namely when participants have already explored both sides of the picture. That is, the FEF-tDCS effect emerged when exploration is more likely to be driven by internal, top-down, mechanisms, as compared to the initial phases. In future studies, more structured goal-directed tasks (such as a visual search paradigm) could be implemented within the FVE [35] to verify this hypothesis.

Previous works applying tDCS over the right FEF have investigated attentional orienting measuring the saccadic latency towards peripheral targets; mixed findings were obtained with such paradigms [27,28,29]. For instance, Kanai and colleagues [27] found a contralateral benefit during anodal tDCS, while Reteig and colleagues [28] found no effects of either anodal or cathodal FEF-tDCS delivered during the task. Diana and colleagues [29] found a significant decrease of saccadic latency directed towards left and right targets brought about 10 min of tDCS applied over both the right PPC and the right FEF, but no effects on attentional disengagement. The involvement of FEF in overt attention assessed with an FVE task was also investigated by Cazzoli et al. [32]: low-frequency repetitive TMS over the right FEF reduced the exploration times in both hemifields, in particular at the periphery of the pictures. However, the authors did not explore the temporal dimension (i.e., the viewing time) as done in the present study.

## 4. General Discussion

The present study investigated the effect of anodal conventional tDCS and HD-tDCS delivered offline over two key areas of the frontoparietal attention network, namely the PPC and the FEF of the right hemisphere. Unlike the majority of previous studies, we employed a more ecological paradigm to measure visuospatial orienting, the FVE task, a computerized task in which participants are requested to freely explore pictures of everyday life. 

Overall, we observed a small leftward shift of the gaze position, after the delivery of conventional tDCS over the right PPC when the pre- and post-stimulation performances were compared (Experiment 1). However, when the baseline performance was used to estimate post-tDCS effects, no modulation of attentional orienting emerged for both frontal and parietal stimulations. Moreover, the analysis of the viewing time suggested no effect of tDCS. Even the more focal HD-tDCS (Experiment 2) was ineffective in modulating visuospatial asymmetries, neither by comparing the pre- and post-stimulation performance nor when the baseline performance was taken into consideration. However, by looking at the temporal pattern of visuospatial exploration, a tDCS-induced leftward shift emerged after the stimulation of FEF with HD-tDCS; this effect emerged towards the end of pictures exploration, around 6 s.

These results do not provide definitive conclusions about the effectiveness of offline anodal tDCS over the right FEF or the right PPC on visuospatial orienting, suggesting that the direction of the neuromodulation effects depends on different factors. The choice of the statistical approach (i.e., comparison between baseline and post-tDCS performance vs. controlling for individual baseline performance) was the main factor influencing the results. Indeed, in line with the literature about the state-dependency of tDCS effects [9,57,59], studying the impact of the baseline level of performance highlights the variability of tDCS effects, both by “correcting” post-stimulation effects (thus preventing from “false positives”) and by showing under which circumstance tDCS is most effective (thus preventing from “false negatives” from group-averaged analysis).

A second factor affecting tDCS modulation of free visual exploration is related to the time window of the analysis: we indeed found that time-dependent effects interact with the spatial aspects. This evidence suggests that fine-grained methods, such as the present spatiotemporal approach, may reveal subtle, but significant, effects of neuromodulation, not detectable with gross measures such as the analysis of overall reaction time or response accuracy, at least in the healthy population. On the other hand, this could also imply that, under some experimental conditions, or with respect to some cognitive domains, tDCS effects on healthy human performance could be negligible [6].

Our findings are also of relevance with respect to the putative advantage of using more focal electric stimulations. A lower current spread by using HD electrodes could diminish the effects variability of the conventional tDCS, thus inducing more reliable behavioral changes. This was not the case in the present study, where we found mixed-to-null effects after conventional tDCS of the right PPC, and a very small, but viewing time-dependent, effect after HD-tDCS when delivered over the right FEF, but not over PPC. Overall, these results do not support a substantial advantage of HD-tDCS over the conventional tDCS, at least for modulating overt visuospatial attention. Rather, with our paradigm, a focal electrical stimulation seems to induce different behavioral effects than the standard tDCS. In this regard, Masina and co-workers [57] found different electrophysiological changes in EEG frequency bands linked to the focality of the stimulation, showing that alpha power was selectively affected by HD-tDCS, whereas beta power was modulated by conventional tDCS.

It should be noted that, in our two experiments, the conventional tDCS and the HD-tDCS electrodes differed both for shape and size, although similar circular electrodes are available for both techniques [57]. With respect to the size, the reduced area of the target electrode of the HD-tDCS (4.9 vs. 25 cm^2^ of the conventional tDCS electrode) also implies an increase of current density (i.e., 0.2 vs. 0.04 mA/cm^2^) along with a reduced strength of the electrical field (see the simulation shown in Figure 1), in turn, possibly inducing different patterns of neurophysiological effects. Indeed, the behavioral and physiological effects of different current intensities (e.g., 1 vs. 2 mA) are directly linked to current density [61,62,63,64]. Although some works showed enhanced behavioral effects with higher intensities (e.g., 2 vs. 1 mA HD-tDCS [64]), other studies showed more robust effects with lower current intensity (1 vs. 2 mA [65,66]), and even differences in the effect direction, with cathodal tDCS at 2 mA increasing motor cortex excitability, but decreasing it at 1 mA [61]. Therefore, the focality of stimulation is only one of the factors shaping the complexity of tDCS outcomes.

Additionally, whereas conventional tDCS electrodes are often placed on both hemispheres, the HD-tDCS electrodes are typically confined to one hemisphere. These different montages likely affect the excitability of different intra- and inter-hemispheric networks, a non-trivial aspect in the field of visuospatial orienting [17]. Future studies are required to compare the effect of conventional tDCS and HD-tDCS from the perspective of connectivity of task-related brain networks, for example, by means of TMS-EEG [5,67,68].

Another limit of the present study is the absence of a within-subject design that would have allowed a direct comparison between HD-tDCS and conventional tDCS [57], along with the intrinsic differences between Experiments 1 and 2 with respect to sample sizes and to the different number of stimuli presented during the FVE task. These methodological differences may have further impacted the intrinsic inter-individual variability stemming from tasks and from the two neuromodulation techniques, but they also have precluded a between-experiment comparison. Therefore, we cannot exclude the possibility that all these factors could have played a role in our findings.

A final consideration regards the present tDCS protocol. Here we applied tDCS offline, at rest, in the absence of a concurrent task, using a stimulation protocol proved to be effective in modulating visuospatial attention [13]. However, the behavioral and neurophysiological effects of tDCS are state-dependent [8,69], with target networks more effectively modulated when they are engaged in a task. Instead, offline neuromodulation protocols seem to primarily affect the Default Mode Network [70]. Future investigations should explore whether the online approach could be more appropriate to reduce variability, giving rise to more reliable effects at the FVE task.

All these reflections will be helpful to design future neuromodulation studies on visuospatial attention, even for rehabilitation purposes. So far, very different protocols with conventional tDCS have been used for the treatment of the syndrome of unilateral spatial neglect [71], while the effectiveness of HD-tDCS still needs to be explored.

In conclusion, our work provides a novel insight into the usefulness of tDCS for modulating visual field exploration and attentional orienting in an ecological setting, also encouraging reflections and future research on differences and advantages of conventional tDCS and HD-tDCS. The present results highlight the complex relationship among target areas, focality of stimulation, spatiotemporal aspects of deployment of attention, and the role of the individual baseline performance in shaping tDCS effects.

## Figures and Tables

**Figure A1:**
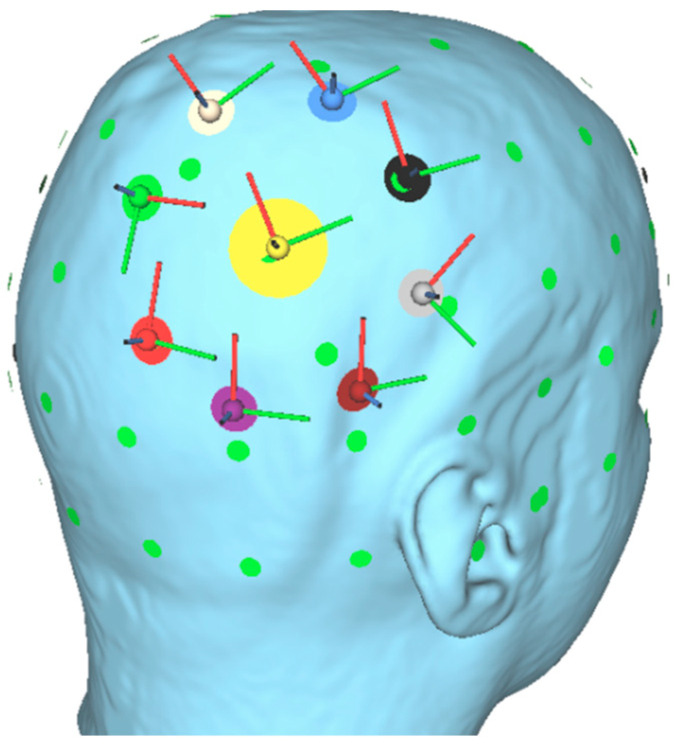
Example of electrodes configuration for the simulation of PPC HD-tDCS. In yellow, the target electrode. The colored, small, electrodes around the target represent an approximation of the ring-shaped return electrode.

**Figure A2:**
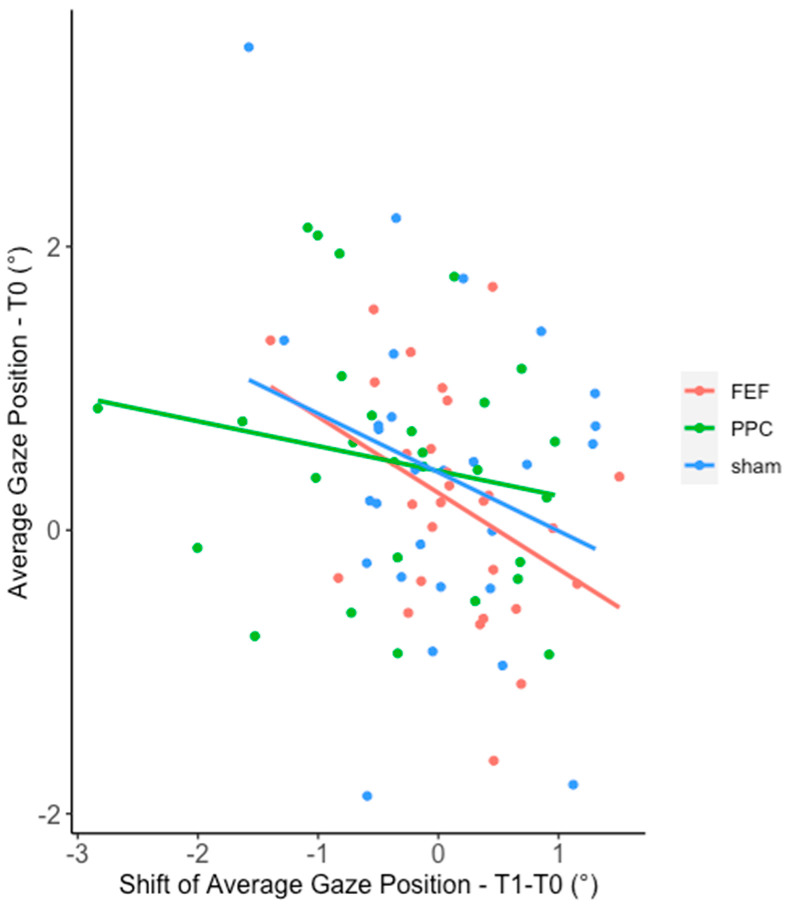
Regression lines of the shift of average gaze position (T1–T0) on the baseline performance (T0) for Frontal Eye Field (FEF), Posterior Parietal Cortex (PPC), and sham stimulation.

## Data Availability

Dataset, pictures, and their respective saliency values are available at: https://osf.io/4sdwr/ (accessed on 7 November 2021).

## Appendix A. Electric Field Simulation with SimNIBS 3.2.4

Current distribution was simulated on the head model provided with the software (i.e., “Ernie”). First, MNI coordinates of target areas were transformed into the subject space by means of a dedicated MATLAB function (mni2subject_coords): 38.3, −70.2, 59.9 for PPC and 20.8, −17.5, 82 for FEF. The software auto-adjusted these values, thus resulting in the following: 50.1, −82.74, 66.82 for PPC and 34.76, −12.11, 107.4 for FEF.

For the conventional tDCS montage, the target electrode was assigned a current value of +1 mA, electrode size = 5 cm × 5 cm, electrode thickness = 1 mm, sponge thickness = 2 mm, sponge size = 6 cm × 5 cm. The reference electrode was located over the contralateral supraorbital position at −31.46, 81.07, 48.78 for both PPC and FEF simulation. It was assigned a current value of −1 mA, electrode size = 7 cm × 5 cm, electrode thickness = 1 mm, sponge thickness = 2 mm; sponge size = 8 cm × 6 cm.

For the HD-tDCS montage, we used the same coordinates and current intensity for the target electrode, but we specified: elliptical electrode size = 2.5 cm × 2.5 cm, electrode thickness = 1 mm, gel thickness = 1 mm. As for the reference ring electrode, in the absence of a proper setup, we simulated eight small circular electrodes (elliptical electrode size = 1.25 cm × 1.25 cm-corresponding to the width of the ring-, electrode thickness = 1 mm, gel thickness = 1 mm), positioned around an imaginary circle of 7.5 cm of diameter–the inner diameter of the ring electrode—centered to the target electrode position. See Figure A1 for an example. Each of the eight small electrodes was assigned a current value of −0.125 mA, resulting in a total current of −1 mA.

## Appendix B. Experiment 1—Conventional tDCS

### Appendix B.1. tDCS-Related Sensations and Sham Blinding

At the end of each tDCS session, we administered a seven-item questionnaire (adapted from [46]) to evaluate the side-effects of tDCS. Participants were asked to report whether they felt (1) itching, (2) pain, (3) burning, (4) heat, (5) pinching, (6) metallic taste, or (7) fatigue, rating the intensity of their sensations for each item on a five-point scale (i.e., 0 = absent, 1 = Mild, 2 = Moderate, 3 = Considerable, 4 = Strong). Moreover, they had to indicate when the feeling/discomfort began, when it stopped, where it was localized, and whether it affected their performance. The reported sensations, their frequencies, and intensities can be found in Table A1.

**Table A1:** tDCS-related sensations, their frequency and most reported intensity after frontal eye field (FEF), posterior parietal cortex (PPC), and sham stimulations.

Item	FEF		PPC		Sham	
	*N*	Most Reported	*N*	Most Reported	*N*	Most Reported
Itching	23	Mild	25	Mild	20	Mild
Pain	2	Mild, Moderate	2	Mild	0	
Burning	18	Mild	17	Mild	12	Mild
Heat	11	Mild	11	Mild	12	Mild
Pinching	25	Mild	24	Mild	18	Mild
Metallic Taste	1	Moderate	0		0	
Fatigue	8	Moderate	5	Mild	5	Mild

Overall, all participants localized the sensations on the head and these sensations were never rated as “Strong”. Only one participant reported considerable pinching, burning, and itching after PPC stimulation.

For FEF stimulation, 27 participants experienced these sensations at the beginning of the stimulation, whereas only one participant at the end of the stimulation. Twenty-one participants reported that these sensations stopped quickly or in the middle of the stimulation, whereas for seven participants, these sensations stopped at the end of the stimulation. With respect to the influence on the task, only two participants reported that the feelings may have had a mild effect on the performance. Regarding PPC stimulation, the sensations started at the beginning of the stimulation for all participants and stopped quickly or in the middle of the stimulation for most of them (*N* = 24), whereas for four participants at the end of the stimulation. Concerning the influence of the tDCS side-effects on the task, only one participant reported that felt sensation could have had a mild effect on the performance. For sham stimulation, four participants reported no tDCS-related sensations. Twenty-three participants experienced tDCS side-effects at the beginning of the stimulation, one participant in the middle. For 22 of them, the sensations stopped quickly, whereas for two at the end of the stimulation. No participants reported that tDCS-related sensations could have affected their performance.

Responses to the questionnaire were statistically analyzed to explore differences among sessions. Participants’ total score at the questionnaire (maximum score = 28, indexing that each of the seven questionnaire items was rated as “Strong”, obtaining a score of 4) in each tDCS session was analyzed by means of an LMM analysis with “Stimulation” as a factor; random intercepts were considered for participants. The significance of the fixed effects was evaluated by means of an F-test with Satterthwaite’s method and Bonferroni-corrected post-hoc contrasts were used whenever necessary. A significant difference emerged between tDCS sessions (*F*_254_ = 5.34; *p* = 0.008): as compared to sham tDCS (mean total score = 2.46, SE = 0.4), higher scores were reported for both FEF (mean total score = 4, SE = 0.4; *p* = 0.013) and PPC tDCS (mean total score = 3.82, SE = 0.4; *p* = 0.032), with no differences between them (*p* = 0.9).

A chi-square analysis was performed to test the participants’ ability to discriminate between real and sham stimulation. As shown in the table of contingence (Table A2), whereas most of the participants correctly identified FEF and PPC stimulations as real, sham stimulation was correctly identified by less than half of participants [*χ*^2^(2) = 9.58; *p* = 0.008]. However, only seven participants correctly identified all three stimulations.

**Table A2:** Blinding to conventional sham stimulation.

	FEF		PPC		Sham	
	Count	Adapted Residuals	Count	Adapted Residuals	Count	Adapted Residuals
Correct	23	1.3	21	1.8	11	−3.1
Wrong	5	−1.3	7	−1.8	17	3.1

Detailed Statistics–Random permutation model.

**Table A3:** Cluster of time-bins with mass value >4 for Stimulation and Timepoint.

Factor	Start	End	Cluster Mass	*p* (>Mass)
Stimulation	62	62	4.88	0.491
Timepoint	3	3	5.70	0.73
	19	19	5.3	0.79
	24	24	4.13	0.95
	30	30	5.04	0.83
	44	44	4.25	0.93
	53	53	4.41	0.92

### Appendix B.2. The Relationship between Baseline and the Change in Gaze Position

An LMM was run with the shift of the mean gaze position after tDCS (i.e., T1–T0) as the dependent variable; fixed effects were tested for Stimulation (FEF, PPC, and sham), with the baseline performance (i.e., mean gaze position at T0), and the interaction between Stimulation and the baseline. Random intercepts were allowed for participants. The significance of the fixed effects was evaluated by means of an F-test with Satterthwaite’s method, and Bonferroni-corrected post-comparisons were used. The interaction between the baseline performance and Stimulation was decomposed by analyzing the simple effects.

The model yielded no effect of Stimulation (*F*_260.34_ = 0.19; *p* = 0.825) and of baseline by Stimulation interaction (*F*_260.38_ = 0.2; *p* = 0.82), but a significant effect of the baseline (*F*_166.04_ = 7.15; *b* = −0.27; *p* = 0.009), indicating a negative association with the shift of gaze position, which is shown in Figure A2.

## Appendix C. Experiment 2—HD-tDCS

### Appendix C.1. HD-tDCS-Related Sensations and Sham-Blinding

Similar to Experiment 1, at the end of each tDCS session, a seven-item questionnaire evaluating potential side-effects of tDCS was administered. The reported sensations, their frequencies, and intensities can be found in Table A4.

**Table A4:** HD-tDCS-related sensations, their frequency, and the most reported intensity after frontal eye field (FEF), posterior parietal cortex (PPC), and sham stimulations.

Item	FEF		PPC		Sham	
	*N*	Most Reported	*N*	Most Reported	*N*	Most Reported
Itching	17	Mild	16	Moderate	15	Mild
Pain	9	Mild	8	Mild	7	Mild
Burning	15	Mild	14	Mild	12	Mild
Heat	8	Mild	13	Mild	8	Mild
Pinching	20	Mild	18	Mild	17	Mild
Metallic Taste	0		1	Mild	0	
Fatigue	4	Mild	5	Mild	7	Mild

All participants localized the sensations on the head and these sensations were never rated as “Strong”. Only one participant reported “Considerable” burning and heat after FEF stimulation.

For FEF stimulation, all participants experienced tDCS-related sensations at the beginning of the stimulation. Nineteen participants reported that these sensations stopped quickly or in the middle of the stimulation, whereas for three participants they stopped at the end of the stimulation. Five participants reported that tDCS-related sensations had a mild effect on their performance. Regarding PPC stimulation, the sensations started at the beginning of the stimulation for all participants and stopped quickly or in the middle of the stimulation for most of them (*N* = 18), whereas for three participants these sensations stopped at the end of the stimulation. One participant did not report any sensation. Three participants reported that the feelings may have had a mild effect on the performance. For sham stimulation, one participant reported no feelings. Twenty participants experienced these sensations at the beginning of the stimulation, one participant at the end. For all participants, the sensations stopped quickly. Four participants reported that tDCS-related sensations could have influenced their performance. LMM analyses conducted on the questionnaire total scores (see Appendix B) showed a significant difference between stimulations (F_242_ = 3.7; *p* = 0.033): PPC HD-tDCS was associated with more frequent and intense sensations (mean total score = 4.68, SE = 0.49) as compared to sham HD-tDCS (total score = 3.36, SE = 0.49; *p* = 0.048). No significant differences emerged between PPC and FEF HD-tDCS (mean total score = 4.5, SE = 0.49; *p* = 0.1) or between FEF and sham HD-tDCS (*p* = 0.109).

With respect to the blinding to sham stimulation, the chi-square analysis indicated no significant association between the type of stimulation and the correctness of the guess [*χ*^2^(2) = 5.96; *p* = 0.051] (see Table A5). Only three participants correctly identified the three stimulation.

**Table A5:** Blinding to HD sham stimulation.

	FEF		PPC		Sham	
	Count	Adapted Residuals	Count	Adapted Residuals	Count	Adapted Residuals
Correct	14	1.3	14	1.8	7	−3.1
Wrong	8	−1.3	8	−1.8	15	3.1

### Appendix C.2. “HD-Baseline” Model–Detailed Statistics

**Table A6:** “HD-baseline-model”-Type III Analysis of Variance Table with Satterthwaite’s method.

Factor	Sum Sq	MeanSq	NumDF	DenDF	F Value	*p*
Stim	482.22	241.11	2	101.96	0.39	0.676
Timepoint	120.33	120.33	1	95.5	0.2	0.659
Baseline	808.69	808.69	1	117.91	1.32	0.254
Stim*Timepoint	2779.49	1389.74	2	95.5	2.27	0.11
Stim*Baseline	507.20	253.60	2	101.96	0.41	0.663
Timepoint*baseline	127.11	127.11	1	95.5	0.21	0.65
3-ways interaction	2885.26	1442.63	2	95.5	2.35	0.101

## References

[1] Lefaucheur J.P., Antal A., Ayache S.S., Benninger D.H., Brunelin J., Cogiamanian F., Cotelli M., De Ridder D., Ferrucci R., Langguth B. (2017). Evidence-Based Guidelines on the Therapeutic Use of Transcranial Direct Current Stimulation (TDCS). Clin. Neurophysiol..

[2] Nitsche M.A., Paulus W. (2000). Excitability Changes Induced in the Human Motor Cortex by Weak Transcranial Direct Current Stimulation. J. Physiol..

[3] Nitsche M.A., Paulus W. (2001). Sustained Excitability Elevations Induced by Transcranial DC Motor Cortex Stimulation in Humans. Neurology.

[4] Monte-Silva K., Kuo M.F., Hessenthaler S., Fresnoza S., Liebetanz D., Paulus W., Nitsche M.A. (2013). Induction of Late LTP-Like Plasticity in the Human Motor Cortex by Repeated Non-Invasive Brain Stimulation. Brain Stimul..

[5] Romero Lauro L.J., Rosanova M., Mattavelli G., Convento S., Pisoni A., Opitz A., Bolognini N., Vallar G. (2014). TDCS Increases Cortical Excitability: Direct Evidence from TMS–EEG. Cortex.

[6] Horvath J.C., Forte J.D., Carter O. (2015). Quantitative Review Finds No Evidence of Cognitive Effects in Healthy Populations From Single-Session Transcranial Direct Current Stimulation (TDCS). Brain Stimul..

[7] Minarik T., Berger B., Althaus L., Bader V., Biebl B., Brotzeller F., Fusban T., Hegemann J., Jesteadt L., Kalweit L. (2016). The Importance of Sample Size for Reproducibility of TDCS Effects. Front. Hum. Neurosci..

[8] Fertonani A., Miniussi C. (2017). Transcranial Electrical Stimulation: What We Know and Do Not Know about Mechanisms. Neuroscientist.

[9] Learmonth G., Thut G., Benwell C.S.Y., Harvey M. (2015). The Implications of State-Dependent TDCS Effects in Aging: Behavioural Response Is Determined by Baseline Performance. Neuropsychologia.

[10] Datta A., Bansal V., Diaz J., Patel J., Reato D., Bikson M. (2009). Gyri-Precise Head Model of Transcranial Direct Current Stimulation: Improved Spatial Focality Using a Ring Electrode versus Conventional Rectangular Pad. Brain Stimul..

[11] Bortoletto M., Rodella C., Salvador R., Miranda P.C., Miniussi C. (2016). Reduced Current Spread by Concentric Electrodes in Transcranial Electrical Stimulation (TES). Brain Stimul..

[12] Kuo H.I., Bikson M., Datta A., Minhas P., Paulus W., Kuo M.F., Nitsche M.A. (2013). Comparing Cortical Plasticity Induced by Conventional and High-Definition 4 × 1 Ring TDCS: A Neurophysiological Study. Brain Stimul..

[13] Sparing R., Thimm M., Hesse M.D., Küst J., Karbe H., Fink G.R. (2009). Bidirectional Alterations of Interhemispheric Parietal Balance by Non-Invasive Cortical Stimulation. Brain.

[14] Bolognini N., Olgiati E., Rossetti A., Maravita A. (2010). Enhancing Multisensory Spatial Orienting by Brain Polarization of the Parietal Cortex. Eur. J. Neurosci..

[15] Loftus A.M., Nicholls M.E.R. (2012). Testing the Activation–Orientation Account of Spatial Attentional Asymmetries Using Transcranial Direct Current Stimulation. Neuropsychologia.

[16] Kinsbourne M. (1987). Mechanisms of Unilateral Neglect. Adv. Psychol..

[17] Corbetta M., Shulman G.L. (2002). Control of Goal-Directed and Stimulus-Driven Attention in the Brain. Nat. Rev. Neurosci..

[18] Thiebaut de Schotten M., Dell’Acqua F., Forkel S., Simmons A., Vergani F., Murphy D.G.M., Catani M. (2011). A Lateralized Brain Network for Visuo-Spatial Attention. Nat. Preced..

[19] Giglia G., Mattaliano P., Puma A., Rizzo S., Fierro B., Brighina F. (2011). Neglect-like Effects Induced by TDCS Modulation of Posterior Parietal Cortices in Healthy Subjects. Brain Stimul..

[20] Ball K., Lane A.R., Smith D.T., Ellison A. (2013). Site-Dependent Effects of TDCS Uncover Dissociations in the Communication Network Underlying the Processing of Visual Search. Brain Stimul..

[21] Roy L.B., Sparing G., Fink G.R., Hesse M.D. (2015). Modulation of Attention Functions by Anodal TDCS on Right PPC. Neuropsychologia.

[22] Corbetta M., Akbudak E., Conturo T.E., Snyder A.Z., Ollinger J.M., Drury H.A., Linenweber M.R., Petersen S.E., Raichle M.E., Van Essen D.C. (1998). A Common Network of Functional Areas for Attention and Eye Movements. Neuron.

[23] Grosbras M.H., Laird A.R., Paus T. (2005). Cortical Regions Involved in Eye Movements, Shifts of Attention, and Gaze Perception. Hum. Brain Mapp..

[24] De Haan B., Morgan P.S., Rorden C. (2008). Covert Orienting of Attention and Overt Eye Movements Activate Identical Brain Regions. Brain Res..

[25] Li H.H., Hanning N.M., Carrasco M. (2021). To Look or Not to Look: Dissociating Presaccadic and Covert Spatial Attention. Trends Neurosci..

[26] Casteau S., Smith D.T. (2020). Covert Attention beyond the Range of Eye-Movements: Evidence for a Dissociation between Exogenous and Endogenous Orienting. Cortex.

[27] Kanai R., Muggleton N., Walsh V. (2012). Transcranial Direct Current Stimulation of the Frontal Eye Fields during Pro- and Antisaccade Tasks. Front. Psychiatry.

[28] Reteig L.C., Knapen T., Roelofs F.J.F.W., Ridderinkhof K.R., Slagter H.A. (2018). No Evidence That Frontal Eye Field TDCS Affects Latency or Accuracy of Prosaccades. Front. Neurosci..

[29] Diana L., Pilastro P., Aiello E.N., Eberhard-Moscicka A.K., Müri R.M., Bolognini N. (2021). Saccades, Attentional Orienting and Disengagement: The Effects of Anodal TDCS over Right Posterior Parietal Cortex (PPC) and Frontal Eye Field (FEF). Eye Track. Res. Appl. Symp..

[30] Cazzoli D., Wurtz P., Müri R.M., Hess C.W., Nyffeler T. (2009). Interhemispheric Balance of Overt Attention: A Theta Burst Stimulation Study. Eur. J. Neurosci..

[31] Chiffi K., Diana L., Hartmann M., Cazzoli D., Bassetti C.L., Müri R.M., Eberhard-Moscicka A.K. (2021). Spatial Asymmetries (“Pseudoneglect”) in Free Visual Exploration—Modulation of Age and Relationship to Line Bisection. Exp. Brain Res..

[32] Paladini R.E., Wyss P., Kaufmann B.C., Urwyler P., Nef T., Cazzoli D., Nyffeler T., Müri R.M. (2019). Re-Fixation and Perseveration Patterns in Neglect Patients during Free Visual Exploration. Eur. J. Neurosci..

[33] Delazer M., Sojer M., Ellmerer P., Boehme C., Benke T. (2018). Eye-Tracking Provides a Sensitive Measure of Exploration Deficits After Acute Right MCA Stroke. Front. Neurol..

[34] Kaufmann B.C., Cazzoli D., Pflugshaupt T., Bohlhalter S., Vanbellingen T., Müri R.M., Nef T., Nyffeler T. (2020). Eyetracking during Free Visual Exploration Detects Neglect More Reliably than Paper-Pencil Tests. Cortex.

[35] Nuthmann A., Matthias E. (2014). Time Course of Pseudoneglect in Scene Viewing. Cortex.

[36] Hartmann M., Sommer N.R., Diana L., Müri R.M., Eberhard-Moscicka A.K. (2019). Further to the Right: Viewing Distance Modulates Attentional Asymmetries (‘Pseudoneglect’) during Visual Exploration. Brain Cogn..

[37] Cazzoli D., Jung S., Nyffeler T., Nef T., Wurtz P., Mosimann U.P., Müri R.M. (2015). The Role of the Right Frontal Eye Field in Overt Visual Attention Deployment as Assessed by Free Visual Exploration. Neuropsychologia.

[38] Brysbaert M., Stevens M. (2018). Power Analysis and Effect Size in Mixed Effects Models: A Tutorial. J. Cogn..

[39] Oldfield R.C. (1971). The Assessment and Analysis of Handedness: The Edinburgh Inventory. Neuropsychologia.

[40] Bikson M., Grossman P., Thomas C., Louis Zannou A., Jiang J., Adnan T., Mourdoukoutas A.P., Kronberg G., Truong D., Boggio P. (2016). Safety of Transcranial Direct Current Stimulation: Evidence Based Update 2016. Brain Stimul..

[41] Thair H., Holloway A.L., Newport R., Smith A.D. (2017). Transcranial Direct Current Stimulation (TDCS): A Beginner’s Guide for Design and Implementation. Front. Neurosci..

[42] Itti L., Koch C., Niebur E. (1998). A Model of Saliency-Based Visual Attention for Rapid Scene Analysis. IEEE Trans. Pattern Anal. Mach. Intell..

[43] Koessler L., Maillard L., Benhadid A., Vignal J.P., Felblinger J., Vespignani H., Braun M. (2009). Automated Cortical Projection of EEG Sensors: Anatomical Correlation via the International 10-10 System. Neuroimage.

[44] Kincade J.M., Abrams R.A., Astafiev S.V., Shulman G.L., Corbetta M. (2005). An Event-Related Functional Magnetic Resonance Imaging Study of Voluntary and Stimulus-Driven Orienting of Attention. J. Neurosci..

[45] Thielscher A., Antunes A., Saturnino G.B. (2015). Field Modeling for Transcranial Magnetic Stimulation: A Useful Tool to Understand the Physiological Effects of TMS?. Proceedings of the Annual International Conference of the IEEE Engineering in Medicine and Biology Society, EMBS.

[46] Fertonani A., Ferrari C., Miniussi C. (2015). What Do You Feel If I Apply Transcranial Electric Stimulation? Safety, Sensations and Secondary Induced Effects. Clin. Neurophysiol..

[47] R Core Team (2021). R: A Language and Environment for Statistical Computing.

[48] Bates D., Mächler M., Bolker B., Walker S. (2015). Fitting Linear Mixed-Effects Models Using Lme4. J. Stat. Softw..

[49] Wickham H. (2016). ggplot2: Elegant Graphics for Data Analysis. Springer-Verlag New York. https://ggplot2.tidyverse.org.

[50] Wickham H., Averick M., Bryan J., Chang W., McGowan L.D., François R., Grolemund G., Hayes A., Henry L., Hester J. (2019). Welcome to the Tidyverse. J. Open Source Softw..

[51] Kuznetsova A., Brockhoff P.B., Christensen R.H.B. (2017). LmerTest Package: Tests in Linear Mixed Effects Models. J. Stat. Softw..

[52] Lenth R. (2020). Emmeans: Estimated Marginal Means, Aka Least-Squares. R Package Version 1.5.0. Means. https://cran.r-project.org/web/packages/emmeans/emmeans.pdf.

[53] Frossard J., Renaud O. (2019). Permutation Tests for Regression, ANOVA, and Comparison of Signals: The Permuco Package. https://www.jstatsoft.org/article/view/v099i15.

[54] RStudio Team (2020). RStudio: Integrated Development for R.

[55] Salvaggio S., Masson N., Andres M. (2019). Eye Position Reflects the Spatial Coding of Numbers During Magnitude Comparison. J. Exp. Psychol. Learn. Mem. Cogn..

[56] Clifton L., Clifton D.A. (2019). The Correlation between Baseline Score and Post-Intervention Score, and Its Implications for Statistical Analysis. Trials.

[57] Masina F., Arcara G., Galletti E., Cinque I., Gamberini L., Mapelli D. (2021). Neurophysiological and Behavioural Effects of Conventional and High Definition TDCS. Sci. Rep..

[58] Maris E., Oostenveld R. (2007). Nonparametric Statistical Testing of EEG- and MEG-Data. J. Neurosci. Methods.

[59] Splittgerber M., Salvador R., Brauer H., Breitling-Ziegler C., Prehn-Kristensen A., Krauel K., Nowak R., Ruffini G., Moliadze V., Siniatchkin M. (2020). Individual Baseline Performance and Electrode Montage Impact on the Effects of Anodal TDCS Over the Left Dorsolateral Prefrontal Cortex. Front. Hum. Neurosci..

[60] Martin A.K., Dzafic I., Ramdave S., Meinzer M. (2017). Causal Evidence for Task-Specific Involvement of the Dorsomedial Prefrontal Cortex in Human Social Cognition. Soc. Cogn. Affect. Neurosci..

[61] Batsikadze G., Moliadze V., Paulus W., Kuo M.-F., Nitsche M.A. (2013). Partially Non-Linear Stimulation Intensity-Dependent Effects of Direct Current Stimulation on Motor Cortex Excitability in Humans. J. Physiol..

[62] Chew T., Ho K.A., Loo C.K. (2015). Inter- and Intra-Individual Variability in Response to Transcranial Direct Current Stimulation (TDCS) at Varying Current Intensities. Brain Stimul..

[63] Esmaeilpour Z., Marangolo P., Hampstead B.M., Bestmann S., Galletta E., Knotkova H., Bikson M. (2018). Incomplete Evidence That Increasing Current Intensity of TDCS Boosts Outcomes. Brain Stimul..

[64] Fiori V., Nitsche M.A., Cucuzza G., Caltagirone C., Marangolo P. (2019). High-Definition Transcranial Direct Current Stimulation Improves Verb Recovery in Aphasic Patients Depending on Current Intensity. Neuroscience.

[65] Papazova I., Strube W., Becker B., Henning B., Schwippel T., Fallgatter A.J., Padberg F., Palm U., Falkai P., Plewnia C. (2018). Improving Working Memory in Schizophrenia: Effects of 1 mA and 2 mA Transcranial Direct Current Stimulation to the Left DLPFC. Schizophr. Res..

[66] Ehrhardt S.E., Filmer H.L., Wards Y., Mattingley J.B., Dux P.E. (2021). The Influence of TDCS Intensity on Decision-Making Training and Transfer Outcomes. J. Neurophysiol..

[67] Pisoni A., Mattavelli G., Papagno C., Rosanova M., Casali A.G., Romero Lauro L.J. (2018). Cognitive Enhancement Induced by Anodal TDCS Drives Circuit-Specific Cortical Plasticity. Cereb. Cortex.

[68] Hill A.T., Rogasch N.C., Fitzgerald P.B., Hoy K.E. (2018). Effects of Single versus Dual-Site High-Definition Transcranial Direct Current Stimulation (HD-TDCS) on Cortical Reactivity and Working Memory Performance in Healthy Subjects. Brain Stimul..

[69] Hill A.T., Rogasch N.C., Fitzgerald P.B., Hoy K.E. (2019). Impact of Concurrent Task Performance on Transcranial Direct Current Stimulation (TDCS)-Induced Changes in Cortical Physiology and Working Memory. Cortex.

[70] Li L.M., Violante I.R., Leech R., Ross E., Hampshire A., Opitz A., Rothwell J.C., Carmichael D.W., Sharp D.J. (2019). Brain State and Polarity Dependent Modulation of Brain Networks by Transcranial Direct Current Stimulation. Hum. Brain Mapp..

[71] Salazar A.P.S., Vaz P.G., Marchese R.R., Stein C., Pinto C., Pagnussat A.S. (2018). Noninvasive Brain Stimulation Improves Hemispatial Neglect After Stroke: A Systematic Review and Meta-Analysis. Arch. Phys. Med. Rehabil..

